# Clinical and molecular characterization of primary hyperoxaluria in Egypt

**DOI:** 10.1038/s41598-022-17980-9

**Published:** 2022-09-23

**Authors:** Neveen A. Soliman, Mohamed A. Elmonem, Safaa M. Abdelrahman, Marwa M. Nabhan, Yosra A. Fahmy, Andrea Cogal, Peter C. Harris, Dawn S. Milliner

**Affiliations:** 1 Department of Pediatrics, Center of Pediatric Nephrology and Transplantation (CPNT), Cairo University, Cairo, Egypt; 2 EGORD, Egyptian Group of Orphan Renal Diseases, Cairo, Egypt; 3 Department of Clinical and Chemical Pathology, Faculty of Medicine, Cairo University, Cairo, Egypt; 4 Egypt Center for Research and Regenerative Medicine (ECRRM), Cairo, Egypt; 5 Division of Nephrology, Departments of Pediatrics and Internal Medicine, Mayo Clinic, Rochester, MN USA; 6 Rare Kidney Stone Consortium (RKSC), Rochester, MN USA

**Keywords:** Genetics, Nephrology

## Abstract

Primary hyperoxaluria (PH) is an autosomal recessive disorder of oxalate metabolism caused by pathogenic variants in either of three genes (*AGXT*, *GRHPR* or *HOGA1*). The study aimed at characterizing the clinical phenotypes as well as the genotypic spectrum of PH in Egypt. We screened 25 Egyptian patients suspected of PH for the three responsible genes by Sanger sequencing. We diagnosed 20 patients from 18 unrelated families, in which the natural history, family history, clinical features and genotypes were evaluated. PH patients were 15 males and 5 females ranging in age from 4 months to 31 years (median 8 years). Fifteen families were consanguineous (83%) and familial clustering was reported in six families (33%). Pathogenic variants in all 40 alleles were in *AGXT*, with none detected in *GRHPR* or *HOGA1*. We detected two novel pathogenic variants c.166-1_172dupGATCATGG (p.Asp58Glyfs*65) and c.766delC (p.Gln256fs*16) and seven previously reported variants in our cohort. This is the first study reporting the genotype of a considerable number of PH1 patients from Egypt. Our detected variants in the *AGXT* gene could form the basis for future genetic counseling and prenatal diagnosis in Egypt and surrounding populations.

## Introduction

Primary hyperoxaluria (PH) is a rare autosomal recessive disease that results in hepatic overproduction and excessive urinary excretion of oxalate which cannot be metabolized. High urinary concentrations of oxalate form complexes with calcium causing recurrent urolithiasis. Calcium oxalate crystals injure the renal tubular epithelium with deposition causing nephrocalcinosis and induce kidney inflammation and fibrosis^1,2^. As the glomerular filtration rate declines due to progressive renal disease, plasma levels of oxalate rise exceeding the renal excretory capacity with subsequent deposition of calcium oxalate in all tissues, particularly bones, joints, retina, heart, vessels, skin, soft tissues, and peripheral and central nervous systems. Oxalosis is therefore associated with pain, disability, unacceptable quality of life and early mortality^3^. In recent years advances in our understanding of the molecular bases of PH have paved the way for the development of new therapeutic strategies. These include substrate-reduction therapies, gene therapy, enzyme administration approaches, colonization with oxalate-degrading intestinal microorganisms, and in PH1, design of pharmacological chaperones^4^.

Primary hyperoxaluria type 1 (PH1) is caused by pathogenic variants in the *AGXT* gene leading to a deficiency of the liver-specific peroxisomal enzyme alanine glyoxylate aminotransferase (AGT), which catalyzes the transamination of glyoxylate to glycine. It is the most common (~ 80%) and most severe form of the three phenotypes of PH. The other phenotypes (PH2 and PH3) are caused by disease causing variants in the *GRHPR* and *HOGA1*, respectively^1–3^. Most clinically relevant variants in *AGXT* gene are missense variants commonly leading to mistargeting of AGT enzyme to the mitochondrion instead of the peroxisome. In some of these variants, vitamin B6 (pyridoxine), which is a cofactor of AGT can partially normalize the enzyme activity^5^.

Although, PH1 accounts for about 0.5% of pediatric end-stage kidney disease (ESKD) in registries from Europe, USA, and Japan it represents up to ~ 10% in Kuwait and ~ 13% in Tunisian ESKD children^6–10^. This is presumably due to the higher rate of consanguineous marriages in the Arab communities.

Primary hyperoxaluria is often missed or misdiagnosed in Egypt due to the lack of routine genetic diagnosis. Furthermore, age at presentation of PH1 is variable with heterogeneous clinical presentation varying from severe infantile onset with failure to thrive and renal impairment down to solitary or multiple stones in adult patients and could be even asymptomatic^11^. The discovery of the mRNA interfering drug lumasiran and its recent approval in the USA and EU as the first specific therapy for PH1 has given the early detection and genetic confirmation of PH1 patients much more importance^12^.

In the current study, we aimed to evaluate the clinical and mutational spectrum of 20 patients with primary hyperoxaluria for the first time in Egypt, aiming at finding phenotypic landmarks and genetic hotspots that may facilitate diagnosis and promote genetic counseling for this relatively commonly encountered disease in Egypt.

## Subjects and methods

### Patients

Patients presenting to Cairo University Children's Hospital over the period June 2014- December 2015 with nephrolithiasis of two or more radiopaque stones and/or nephrocalcinosis, either cortical, medullary, or corticomedullary and patients who presented before ESKD coupled with increased urinary oxalate excretion were included in the current study as presumable PH patients (Total number 25). The study was conducted in accordance with the declaration of Helsinki for studies involving human subjects and approved by the institutional review board at Mayo Clinic, Rochester, Minnesota (IRB 17-005513). Written informed consents were obtained from all study participants and/or their legal guardians before recruitment.

The clinical records, imaging studies and pathology reports of all patients with a diagnosis of radiopaque nephrolithiasis and/or nephrocalcinosis were reviewed. Demographic and clinical data were recorded including family history, age of presentation and diagnosis, pattern of clinical presentation in terms of symptoms and findings including the characterization of renal phenotype as well as extra-renal manifestations if any. Estimated glomerular filtration rate (eGFR) was calculated by the Schwartz formula in children < 18 years^13^, and the CKD-EPI formula for patients > 18 years^14^.

Abdominal ultrasonographic examination was performed to categorize the renal phenotype through determining kidney size, echogenicity, and cortico-medullary differentiation. Also site, size, and number of urinary tract stones were determined.

### Genetic analysis

An EDTA blood sample was obtained from patients consenting to molecular testing for DNA isolation. DNA extraction was performed by standard salting out technique as previously described^15^. Direct sequencing was performed of all coding exons and exon–intron boundaries of the involved genes (*AGXT*: NM_000030.2, *GRHPR*: NM_012203.1, *HOGA1*: NM_138413.3) using M13 tailed primers as described previously^16^. DNA sequence chromatograms were analyzed using Mutation Surveyor v4.06 (SoftGenetics, Park Forest, PA, USA). ACMG guidelines for the evaluation of variant pathogenicity were followed^17^. Novel variants were confirmed to be segregating properly in parents according to a strict autosomal recessive inheritance model.

## Results

### Clinical features

**Figure 1:**
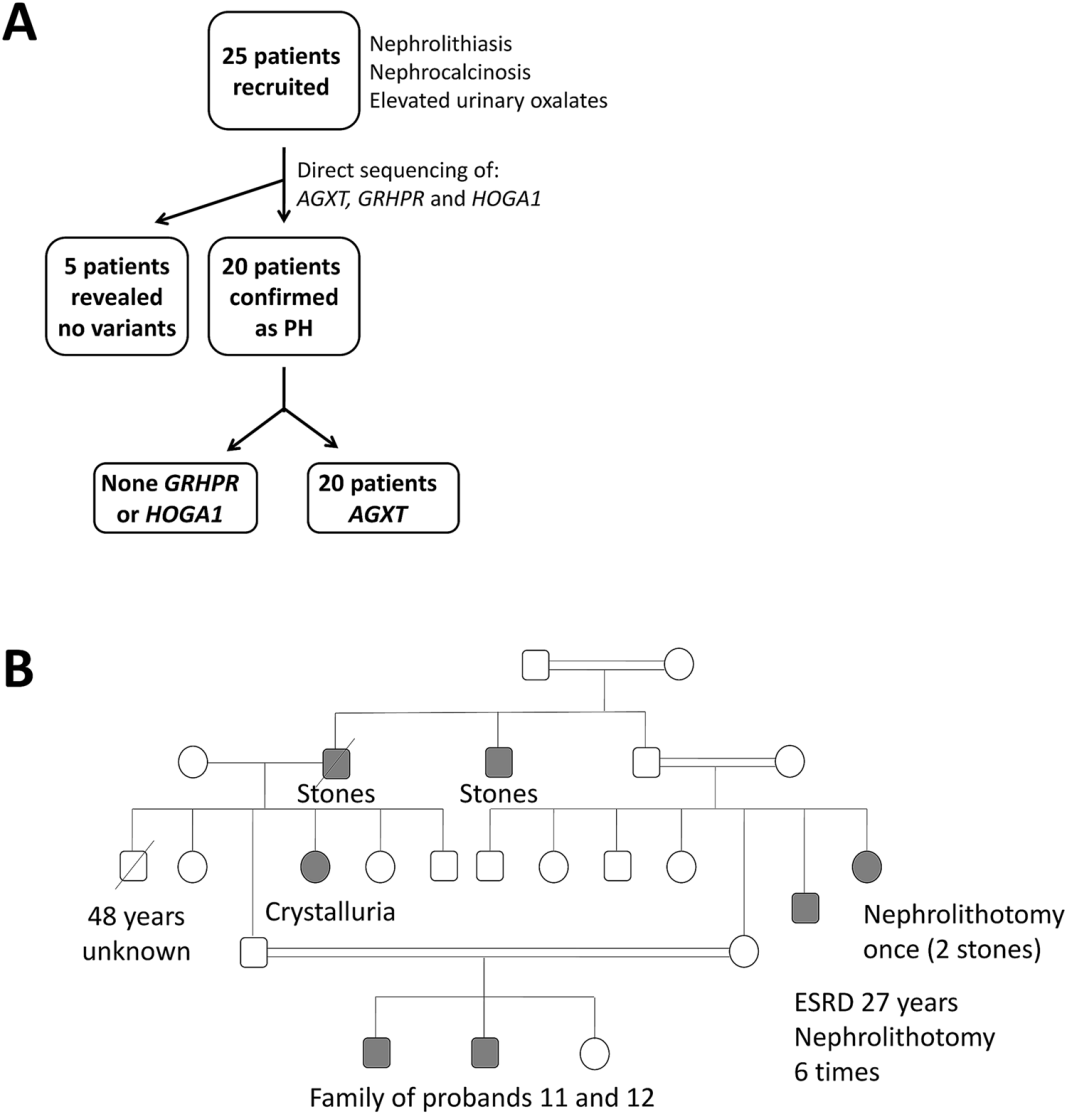
(**A**) A flowchart of patients recruited in the study. (**B**) A pedigree with multiple affected cases in the same family.

We managed to confirm the diagnosis of primary hyperoxaluria genetically in 20 patients out of 25 suspected (Fig. 1A). Those 20 patients belonged to 18 unrelated families. Fifteen of the affected families were consanguineous and familial clustering was reported in 6 families (Fig. 1B shows seven affected individuals in a single Egyptian family pedigree). The median age of onset of symptoms of confirmed PH1 patients was 3 years (range 0.3–16 years) whereas the median age of diagnosis was 8 years (range 0.3–31 years) and diagnostic delay ranged from 0 to 26 years. Males comprised the majority of the studied patients 15/20 with a male to female ratio of 3:1. Notably, growth retardation was the most common presenting symptom reported in 15/20 patients (75%), followed by hematuria in 6/20 (30%), stone passing in 5/20 (25%), and abdominal pain in 4/20 (20%). On the other hand, radiopaque renal stones were the most common presenting finding (90%), followed by ESKD (75%), nephrocalcinosis (60%), post-transplantation recurrence (5%), and sibling screening (5%). (Table 1).

**Table 1:** Demographic and clinical features of Egyptian patients with PH (*N* = 20).

No	Gender	Age at presentation	Age at diagnosis	Consanguinity	Family History	NC	Stones	Location of stones	EGFR at presentation	Liver Kidney Tx/Age (Yr)	Urinary oxalates	Extra-renal manifestations	Outcome
1	M	3	3.5	Pos	No	C&M	Few	Both Kidneys	7	Yes/3.8	N/A	Absent	Post-CLKT living
2	F	7	10	Pos	Yes	N/A	Numerous	Both Kidneys	10	Yes/12	N/A	Absent	Post- CLKT living
3	M	2	2.4	Pos	No	C&M	Few	Both Kidneys	12	No	Normal	Absent	Deceased
4	M	8	12	Pos	Yes	N/A	Numerous	Both Kidneys	5	No	Normal	Absent	Post-KTx living
5	F	0.8	2.4	Pos	Yes	C&M	Not detected	N/A	5	No	N/A	Soft tissues, bones, Retina	Living on dialysis
6	M	7	10.9	Pos	Yes	N/A	Numerous	Both Kidneys	65	No	High	Absent	Living
7	M	0.5	0.5	Pos	Yes	C&M	N/A	N/A	8	No	N/A	Absent	Deceased
8	M	5	6	Pos	No	C&M	Few	Both Kidneys	12	Yes/8	N/A	Absent	Post- CLKT living
9	M	5	12	Pos	No	C&M	Numerous	Both Kidneys	9	No	N/A	Absent	Living on dialysis
10	M	2	10.1	Pos	No	N/A	Few	Both Kidneys	68	No	High	Absent	Living
11*	M	4	10.8	Pos	Yes	N/A	Single	Bladder	7	No	N/A	Absent	Deceased
12*	M	5.7	6.7	Pos	Yes	N/A	Single	Left kidney	82	No	High	Absent	Living
13	F	0.3	0.7	Neg	No	M	Numerous	Both Kidneys	8	No	N/A	Absent	Deceased
14	M	1.2	2.3	Pos	Yes	N/A	Numerous	Both Kidneys	123	No	High	Absent	Living
15	F	0.3	0.3	Pos	Yes	M	N/A	N/A	8	No	High	Absent	Deceased
16**	M	3	29	Neg	Yes	N/A	Numerous	Both Kidneys	12	No	Normal	Absent	Living on dialysis
17**	M	3	29	Neg	Yes	N/A	Numerous	Both Kidneys	59	No	N/A	Absent	Living
18	M	9	10	Pos	No	C/M	Few	Both Kidneys	11	No	Normal	Absent	Living on dialysis
19	M	3	6	Pos	No	C/M	Few	Both Kidneys	9	No	N/A	Absent	Deceased
20	F	15	31	Neg	No	C/M	Numerous	Both Kidneys	6	No	Normal	Absent	Living on dialysis

*C* Cortical, *CLKT* combined liver kidney transplantation, *KTx* kidney transplantation, *M* Medullary, *N/A* not available, *NC* nephrocalcinosis. eGFR was calculated using the Schwartz formula and the CKD-EPI formula for patients < 18 and > 18 years, respectively. High urinary oxalate is defined as > 0.50 mmol oxalate/1.73 m2/day in a 24 h urinary sample. * Siblings, ** Identical twins.

In our study, 3/20 patients were categorized as CKD 2, 1/20 as CKD3, whereas 15/20 patients had already reached ESKD (CKD4/5). CKD staging was based on the classification provided by the Kidney Disease Improving Global Outcome (KDIGO) initiative^18^. Among ESKD patients, six patients were still on regular hemodialysis, six patients died due to dialysis related complications, whereas in the remaining three patients simultaneous combined liver and kidney transplantation was successfully performed with functioning grafts for 2–7 years to date.

### Genetic spectrum

**Figure 2:**
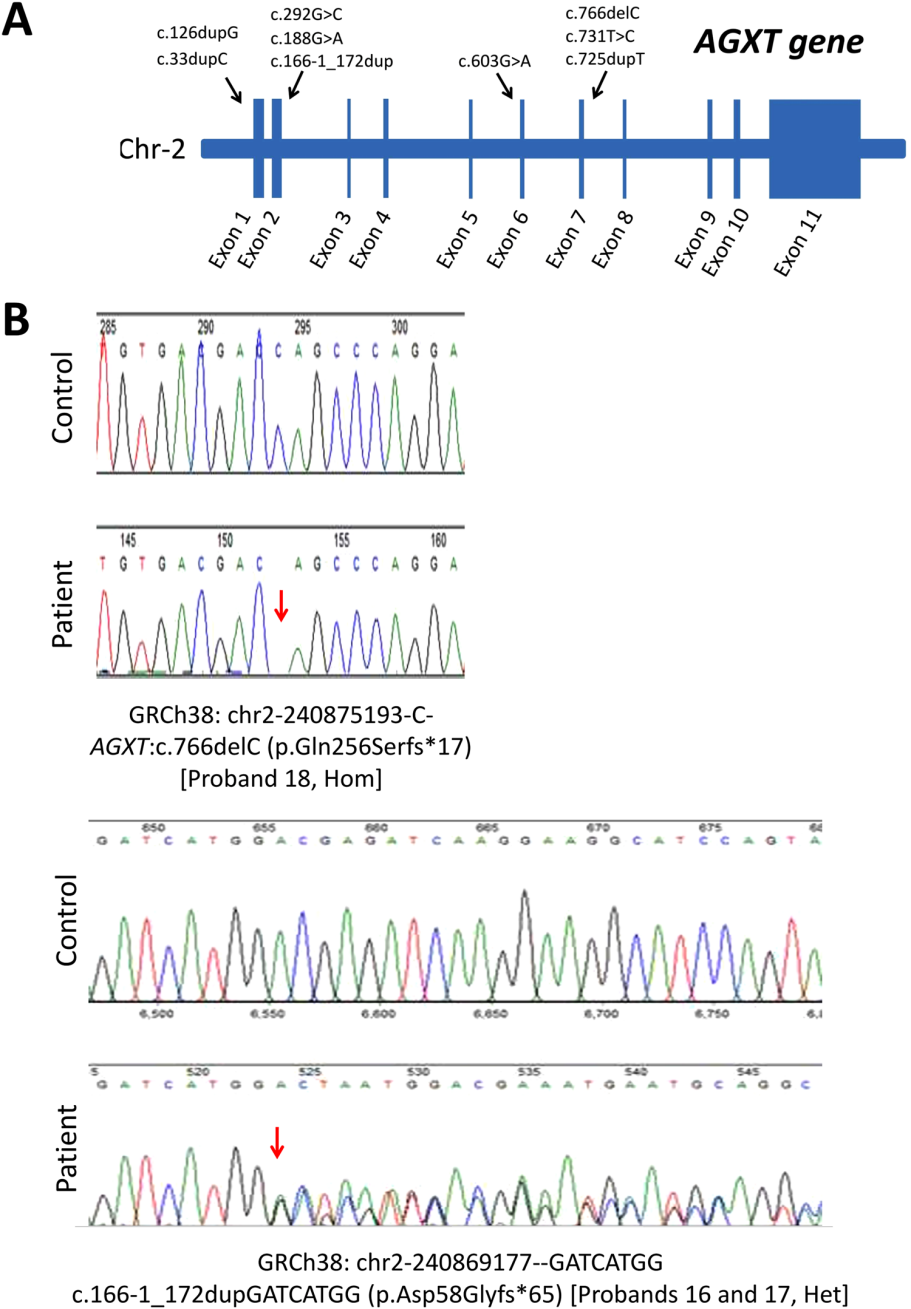
(**A**) A schematic representation of *AGXT* gene exons showing all pathogenic variants detected in Egyptian PH patients. (**B**) Novel variants in the *AGXT* gene in Egyptian patients: c.766delC (p.Gln256Serfs*17) and c.166-1_172dupGATCATGG (p.Asp58Glyfs*65) compared with a normal individual. Het: heterozygous, Hom: homozygous.

All 40 alleles showed pathogenic variants in the *AGXT* gene (Table 2), while none was detected in *GRHPR* or *HOGA1*. Nine pathogenic variants were detected (Fig. 2A) including two novel ones (Fig. 2B). Both were frame shift variants (c.166-1_172dupGATCATGG (p.Asp58Glyfs*65) and c.766delC (p.Gln256Serfs*17)) leading to an abnormal premature termination of the protein. Seven previously reported variants were also detected (c.731 T > C (p.Ile244Thr), c.33dupC (p.Lys12Glnfs*156), c.33delC (p.Lys12Argfs*34), c.126dupG (p.Leu43Alafs*125), c.188G > A (p.Gly63Asp); c.725dupT (p.Asp243Glyfs*12), c.603C > A (p.Asp201Glu) and c.292G > C (p.Asp98His)). All variant nomenclature complies with the guidelines of the human genome variation society (HGVS) and all nine variants were extremely rare in the GnomAD database (population frequency < 0.0001 among 251,428 sequenced individuals). The most common pathogenic variant detected in Egyptian patients was p.Ile244Thr (10/36 mutant familial alleles), which is also the most common pathogenic variant in PH patients from North Africa^19–21^. Novel *AGXT* variants have been submitted to the Leiden Open Variation Database (LOVD) under variant IDs: 0000763697 (patients 16, 17) and 0000763698 (Patients 18).

**Table 2:** *AGXT* mutational spectrum of Egyptian PH1 patients (*N* = 20).

No	Allele 1	Allele 2	Exon	Type of Mutation
1	c.731 T > C (p.Ile244Thr)	c.731 T > C (p.Ile244Thr)	7	Missense
2	c.731 T > C (p.Ile244Thr)	c.731 T > C (p.Ile244Thr)	7	Missense
3	c.731 T > C (p.Ile244Thr)	c.731 T > C (p.Ile244Thr)	7	Missense
4	c.33dupC (p.Lys12Argfs*34)	c.33dupC (p.Lys12Argfs*34)	1	Frameshift
5	c.731 T > C (p.Ile244Thr)	c.731 T > C (p.Ile244Thr)	7	Missense
6	c.126dupG(p.Leu43Alafs*125)	c.126dupG(p.Leu43Alafs*125)	1	Frameshift
7	c.731 T > C (p.Ile244Thr)	c.731 T > C (p.Ile244Thr)	7	Missense
8	c.725dupT (p.Asp243Glyfs*12)	c.725dupT (p.Asp243Glyfs*12)	7	Frameshift
9	c.33dupC (p.Lys12Argfs*34)	c.33dupC (p.Lys12Argfs*34)	1	Frameshift
10	c.126dupG(p.Leu43Alafs*125)	c.126dupG(p.Leu43Alafs*125)	1	Frameshift
11*	c.603C > A (p.Asp201Glu)	c.603C > A (p.Asp201Glu)	6	Missense
12*	c.603C > A (p.Asp201Glu)	c.603C > A (p.Asp201Glu)	6	Missense
13	c.292G > C (p.Asp98His)	c.292G > C (p.Asp98His)	2	Missense
14	c.33dupC (p.Lys12Argfs*34)	c.33dupC (p.Lys12Argfs*34)	1	Frameshift
15	c.188G > A (p.Gly63Asp)	c.188G > A (p.Gly63Asp)	2	Missense
16**	c.166-1_172dupGATCATGG (p.Asp58Glyfs*65) (#)	c.725dupT (p.Asp243Glyfs*12)	2 and 7	Frameshift, Frameshift
17**	c.166-1_172dupGATCATGG (p.Asp58Glyfs*65) (#)	c.725dupT (p.Asp243Glyfs*12)	2 and 7	Frameshift, Frameshift
18	c.766delC (p.Gln256Serfs*17) (#)	c.766delC (p.Gln256Serfs*17) (#)	7	Frameshift
19	c.33dupC (p.Lys12Argfs*34)	c.33dupC (p.Lys12Argfs*34)	1	Frameshift
20	c.603C > A (p.Asp201Glu)	c.603C > A (p.Asp201Glu)	6	Missense

(#) Novel mutations in Egyptian PH1 patients, * Siblings, ** Identical twins.

Of note, four patients had infantile PH1 presenting predominantly with failure to thrive and ESKD during the first year of life (Patients 5, 7, 13 and 15). Unsurprisingly, ultrasonographic imaging of their kidneys demonstrated extensively hyperechogenic kidneys consistent with cortical and medullary nephrocalcinosis. Kidney stones were detected in only one of the four patients with infantile PH1 (Table 1).

The three liver/kidney transplanted patients were 2 males and one female (Patients 1, 2 and 8 in our cohort), who were transplanted at the ages of 3.8, 12, and 8 years, respectively. In all three patients, the identified *AGXT* variants were homozygous in nature. The common North African mutation p.Ile244Thr was detected in 2/3 transplanted patients, whereas c.725dupT was identified in the third patient. All three patients have functioning grafts for 7, 6, and 2 years, respectively.

## Discussion

Primary hyperoxaluria Type 1 is a rare inborn error of metabolism that results in overproduction and excessive urinary excretion of oxalate, leading to recurrent urolithiasis and nephrocalcinosis. PH1 is an autosomal recessive disease, which is particularly prevalent in North Africa and Middle East given the high rate of consanguineous marriages^19,22,23^. The presenting clinical findings in PH1patients in this study were similar to clinical phenotypes described in earlier reports^24,25^. A high rate of consanguinity (83%) and familial clustering (30%) were reported, which is not surprising given prevalent endogamy in the population.

Although PH is an autosomal recessive disorder, a striking male preponderance was noted among the current cohort with a male to female ratio of 3:1. This could be partially explained by the differential treatment by parents towards sick male and female children especially in rural areas where most of our patients come from. This observation has been documented in several developing countries^26^.

Radiopaque renal stones (90%), ESKD (75%), growth retardation (75%), and nephrocalcinosis (60%) were the most common reported clinical features. The majority of our cohort had ESKD at initial presentation which could partly be explained by lack of timely diagnosis. PH1 is a heterogeneous disease with a wide age range of presentations and non-specific symptoms, often leading to an incorrect diagnosis. Notably, 75% of our study patients had ESKD at presentation compared to 33% of Dutch adult and childhood PH1 patients and 27% of Tunisian pediatric patients^25,27^.

Siblings in our study (patients 11 & 12 and patients 16 & 17), although having the same genetic backgrounds, their clinical features varied considerably (Table 1). This is very evident in the eGFR at presentation for the identical twins (patients 16 and 17), who one of them presented with ESKD (eGFR 12), while the second presented simultaneously with an eGFR of 59. This is probably indicative of the effects of other environmental or epigenetic factors on patients' presentation.

To date, around 200 pathogenic variants in *AGXT* have been described^4^. The three most common *AGXT* mutations worldwide; c.508G > A (p.Gly170Arg), c.33dupC (p. Lys12Glnfs*156), and c.731 T > C (p.Ile244Thr), account for approximately 30%, 11%, and 6% of *AGXT* variants, respectively^28^. The *AGXT* mutation p.Ile244Thr is common in North African and Spanish populations^19,21,29^, and was the most common pathogenic variant in our study accounting for 28% of mutant *AGXT* alleles. In contrast, the most common pathogenic variant worldwide p.Gly170Arg was not detected at all in Egyptian patients. This variant is associated with significant residual catalytic activity and immune-reactivity in liver biopsy, and is mostly responsive to vitamin B6 (pyridoxine) therapy^30^. Beneficial effects of pyridoxine, as a chemical chaperone in vitro, have been also demonstrated for other missense mutations such as p.Ile244Thr^25^. We previously reported that 26.7% (4/15) Egyptian PH1 patients were responsive to pyridoxine^22^. Combined liver/kidney transplantation also offered a better survival strategy compared to consecutive transplantation for PH1 patients previously diagnosed in Egypt^31^.

The study has some limitations mainly the absence of the allele frequencies of our detected variants in the general Egyptian population; however, all detected variants were extremely rare in the GnomAD database (population frequency less than 0.0001 in over 250,000 individuals). Furthermore, the two novel variants in our study were both frame shift variants and are predicted to be pathogenic.

In conclusion, PH1 is a devastating disease with clinical heterogeneity that often results in late or overlooked diagnosis; hence ESKD is still a common presentation. This study is the first to describe the genotype of a large number of Egyptian PH1 patients. Promoting awareness, timely diagnosis and proper management while searching for an effective cure are crucial to enhance patient care. *AGXT* pathogenic variants in exon 7 followed by exons 1, 2 and 6 accounted for 100% of mutant alleles in Egyptian patients in our study. This could be extremely important for the genetic diagnostic strategy and for genetic counseling of PH in Egypt and surrounding countries.

## Data Availability

All relevant data generated or analyzed during this study are included in this published article. Any further clinical details are available from the corresponding author on reasonable request. Novel *AGXT* variants have been submitted to the Leiden Open Variation Database (LOVD) under variant IDs: 0000763697 (patients 16, 17) and 0000763698 (Patients 18).
